# LUADpp: an effective prediction model on prognosis of lung adenocarcinomas based on somatic mutational features

**DOI:** 10.1186/s12885-019-5433-7

**Published:** 2019-03-22

**Authors:** Jiaxian Yu, Yueming Hu, Yafei Xu, Jue Wang, Jiajie Kuang, Wei Zhang, Jianlin Shao, Dianjing Guo, Yejun Wang

**Affiliations:** 1 Department of Cell Biology and Genetics, Shenzhen University Health Science Center, Shenzhen, 518060 China; 2 State Key Laboratory of Agrobiotechnology and School of Life Science, The Chinese University of Hong Kong, Shatin, Hong Kong, China; 3 Sehnzhen GenRead Technology Co., Ltd., Shenzhen, 518000 China; 4 Zhejiang Hospital, 12 Lingyin Rd, Hangzhou, 310003 China

**Keywords:** Lung adenocarcinomas, Somatic mutational, Personal medicine, Support vector machine model, Machine learning

## Abstract

**Background:**

Lung adenocarcinoma is the most common type of lung cancers. Whole-genome sequencing studies disclosed the genomic landscape of lung adenocarcinomas. however, it remains unclear if the genetic alternations could guide prognosis prediction. Effective genetic markers and their based prediction models are also at a lack for prognosis evaluation.

**Methods:**

We obtained the somatic mutation data and clinical data for 371 lung adenocarcinoma cases from The Cancer Genome Atlas. The cases were classified into two prognostic groups (3-year survival), and a comparison was performed between the groups for the somatic mutation frequencies of genes, followed by development of computational models to discrete the different prognosis.

**Results:**

Genes were found with higher mutation rates in good (≥ 3-year survival) than in poor (< 3-year survival) prognosis group of lung adenocarcinoma patients. Genes participating in cell-cell adhesion and motility were significantly enriched in the top gene list with mutation rate difference between the good and poor prognosis group. Support Vector Machine models with the gene somatic mutation features could well predict prognosis, and the performance improved as feature size increased. An 85-gene model reached an average cross-validated accuracy of 81% and an Area Under the Curve (AUC) of 0.896 for the Receiver Operating Characteristic (ROC) curves. The model also exhibited good inter-stage prognosis prediction performance, with an average AUC of 0.846 for the ROC curves.

**Conclusion:**

The prognosis of lung adenocarcinomas is related with somatic gene mutations. The genetic markers could be used for prognosis prediction and furthermore provide guidance for personal medicine.

**Electronic supplementary material:**

The online version of this article (10.1186/s12885-019-5433-7) contains supplementary material, which is available to authorized users.

## Background

Lung cancer is the leading cause of cancer death in both more and less developed countries, leading to more than 1,000,000 deaths per year globally [1, 2]. Non–small cell lung cancer (NSCLC) is the most common type of lung cancer while adenocarcinoma (LUAD) is its most common subtype [3, 4]. Despite the dramatic improvement for partial LUAD patients by molecule-targeting therapies developed recently, the conventional chemotherapy remains the first choice for most cases, since most LUADs lack an identifiable driver oncogene or mutation [5–9]. To date, tumor-nodal-metastasis (TNM) stage remains the most important indicator for chemotherapeutic prognosis of patients with LUADs [10]. For more than 1/3 of the cases, however, prognosis could not be correctly predicted by the TNM stage [11–13]. The wide mixture of histologic subtypes also limited the clinical application of histologic classifications [14]. Recently, molecular markers, such as EGFR, ERCC1, RRM1, BRCA1, RET, etc., have been experimentally identified and tested for prognostic prediction [15–17]. However, the number of known molecular markers is still so small that even the combination of them could only give a poor discrimination power generally.

As the sequencing technology advances and the costs fall down, whole-genome sequencing (WGS) is turning to be a cost-effective way to obtain the comprehensive genetic information for tumors and other human complex genetic diseases [18–22]. A list of LUAD related somatic alterations have been identified through WGS and other high-throughput studies [4, 23–25]. A number of molecular makers and pathways have been discovered, which are valuable for their potential actions on diagnosis and molecular classification, or serving as underlined therapeutic targets. The comprehensive genomic and case information appears also attractive for possible prognosis prediction and therefore provide useful guidance for personal medicine. However, it remains difficult to find the most significant genetic features and build a high-effective predictive model for treatment outcomes. To confront the challenges, we collected the large-scale LUAD case data with both genome and clinic information (*n* = 371) from TCGA (The Cancer Genome Atlas) (http://cancergenome.nih.gov), analyzed the somatic mutation difference between the two groups categorized based on the 3-year overall survival, and developed a machine learning model to predict prognosis based on the most significant genetic markers. Through the analysis, we identified a list of genes with different mutation frequencies between different prognosis groups and many were involved in cell-cell adhesion and motility; an absolute majority of the genes showed higher mutation frequencies in the good prognosis group. Support Vector Machine (SVM) models were trained with the gene somatic mutation features, which could well predict the prognosis, much better than the performance of the conventional TNM staging system. The training datasets and models for the treatment outcome prediction of lung carcinoma are freely accessible through the website: http://www.szu-bioinf.org/CPP/LUADpp.

## Methods

### Datasets, stratification, and mutation frequency comparison

The clinical data for the patients with lung adenocarcinomas (LUADs) were downloaded from TCGA (The Cancer Genome Atlas) website. The somatic mutation data between tumor-normal pairs of each LUAD were also downloaded. The mutations causing codon changes, frame-shifts, and premature translational terminations were retrieved for further analysis. For prognosis, the cases were removed that received targeting therapy. Furthermore, only the ones with somatic mutation data and corresponding prognostic follow-up information were recruited. The cases were classified into two categories according to prognosis (‘good’ or ‘poor’) [25]. The ‘good’ prognosis group included the patients surviving through the preset follow-up period while the ‘poor’ group indicated the patients died within the observed period. TNM (tumor-nodal-metastasis) staging system was used for stratification, and for convenience of binary classification, two categories were predefined, ‘early’ (Stage I) and ‘later’ (Other stages). To compare the somatic gene mutation frequency between prognosis groups, a matrix was prepared to record the mutations of all the genes for each case, followed by counting the number of cases with mutations for each gene in each group. A genome-wide rate comparison test (EBT) proposed recently that could balance statistic power and precision was adopted to compare the gene mutation rates [26].

### Feature representation and model training

The top *n* genes with most significant mutation frequency difference were used as the genetic features. For each case *P*_*j*_ (*j* = *1*, *2*, …,*m*_*i*_) belonging to a certain category *C*_*i*_, where *i* equaled to 1 or 0, and *m*_*i*_ represented the total number of cases of the category *C*_*i*_, the genetic features were represented as a binary vector *F*_*j*_ (*g*_*1*_,*g*_*2*_,…,*g*_*n*_) in which *g*_*k*_ (*k* = *1*, *2*, …, *n*) represented the *k*^th^ genetic feature, taking the value of 1 if the corresponding gene was mutated and 0 otherwise. There was an *m*_i_*n matrix for category *C*_*i*_. When stage was used as an additional feature, the size of matrix was enlarged to *m*_i_*(*n* + 1), and the stage feature was also represented in a binary form in the additional column, for which 1 and 0 represented ‘early’ and ‘later’, respectively.

An R package, ‘e1071’, was used for training SVM models using each training dataset (http://cran.r-project.org). During training stage, all four kernels, ‘Radial Base Function (RBF)’, ‘linear’, ‘polynomial’ and ‘sigmoid’, were tested and the parameters were optimized based on a 10-fold cross-validation grid search. The best kernel with optimized parameters was selected for further model training.

### Model performance assessment

A 5-fold cross validation strategy was used in this study. The original feature-represented matrix for each category was randomly split into five parts with identical size. Every four parts of each category were combined and served as a training dataset while the rest one of each category was used for testing and performance evaluation.

Receiver Operating Characteristic (ROC) curve, the area under ROC curve (AUC), Accuracy, Sensitivity and Specificity were utilized to assess the predictive performance. In the following formula, Accuracy denotes the percentage of both positive instances (‘good prognosis’) and negative instances (‘poor prognosis’) correctly predicted. Specificity and Sensitivity represent the true negative and true positive rate respectively, while the default threshold value from ‘e1070’ (0.0) was used to define the Sensitivity and Specificity in the research. An ROC curve is a plot of Sensitivity versus (1 - Specificity) and is generated by shifting the decision threshold. AUC gives a measure of classifier performance.

*Accuracy* = (TP + TN)/(TP + FP + TN + FN), Specificity = TN/(TN + FP), *Sensitivity* = TP/(TP + FN).

### Survival analysis

The follow-up survival information of LUAD cases was annotated. To evaluate the survival of prediction results of each model, all the 5-fold cross-validation testing results were collected and grouped, followed by the survival analysis for each predicted group. Kaplan-Meier overall survival analysis was performed with R survival package (https://cran.r-project.org/). Gehan-Breslow-Wilcoxon test was used to compare the difference of overall survival curves, and the significance level was set as 0.05.

## Results

### Somatic mutation difference between groups with different prognosis

**Fig. 1:**
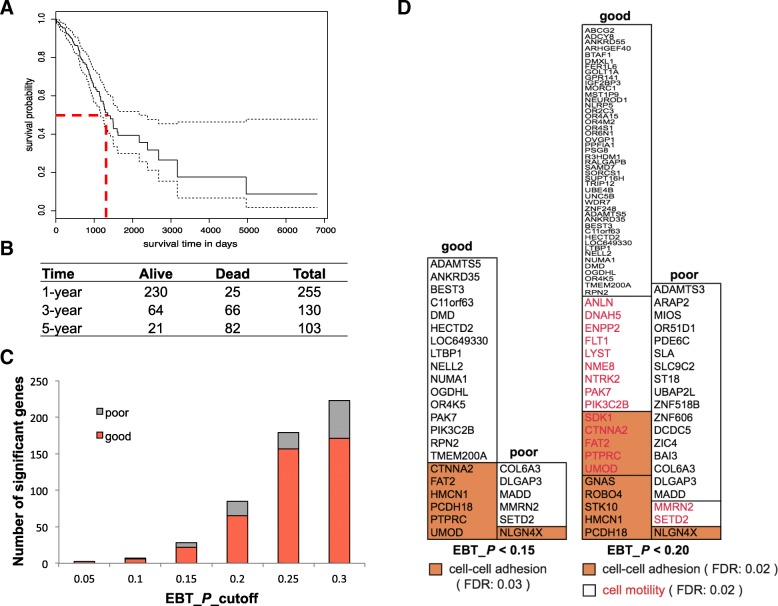
Survival analysis of LUAD cases and comparison of gene somatic mutation rates between different prognosis groups. **a**. Overall survival of LUAD cases. The median survival time was indicated with red dashes. **b**. The survived and dead LUAD cases within 1, 3 and 5 years. **c**. The number of genes with significant mutation rate difference between good and poor prognosis groups at different significance levels. The genes with higher mutation rates in good and poor prognosis groups were shown in red and grey, respectively. **d**. Gene Ontology (GO) biological process enrichment of genes with significant mutation rates between prognosis groups at EBT *p* value < 0.15 and 0.20, respectively. The significantly enriched function clusters were shown in orange background (cell-cell adhesion) or in red (cell motility), respectively (Fisher’s Exact with FDR multiple test correction)

Survival analysis was performed to the LUAD cases with both genome sequencing information and clinical follow-up data (Fig. 1a). The 3-year survival rate was close to 50%, making the cases evenly distributed in two different groups: good (> = 3 years) and poor (< 3 years) prognosis (Fig. 1a, b; Additional file 1: Table S1). Each 3-year group contained not too few samples as in 1-year or 5-year bins, improving the power of further statistical comparisons (Fig. 1b).

To observe the possible association of somatic mutations with LUAD prognosis, gene mutation rate was compared between the two prognostic groups. A newly developed genome-wide rate comparison method, EBT, was adopted to make the comparison instead of multi-test correction based Chi-square or binomial tests, since EBT could improve the statistical power strikingly without apparent loss in precision [26]. The comparison results were shown in Additional file 1: Table S2. Only two genes, ADAMTS5 and PTPRC were found with significant mutation rate difference (EBT, *P* < 0.05). Both genes were with higher mutation rate in good prognosis group (9/64 vs. 0/66 for both). The significance level was relaxed so as to make a further observation of the possible atypical associations of genetics and LUAD prognosis. Interestingly, the good prognostic group always showed much more genes with higher somatic mutation rates (Fig. 1c). Functional enrichment further disclosed that a significant portion of the genes participated in cell-cell adhesion (EBT_*P* < 0.15 gene set: FDR = 0.03; EBT_*P* < 0.20 gene set: FDR = 0.02) and cell motility (EBT_*P* < 0.20 gene set: FDR = 0.02) (Fig. 1d; Additional file 1: Table S2). The cell-cell adhesion and cell motility genes were strikingly enriched in the good prognostic group (Fig. 1D; Additional file 1: Table S2).

### Prognosis prediction of LUAD with somatic gene mutation features

**Fig. 2:**
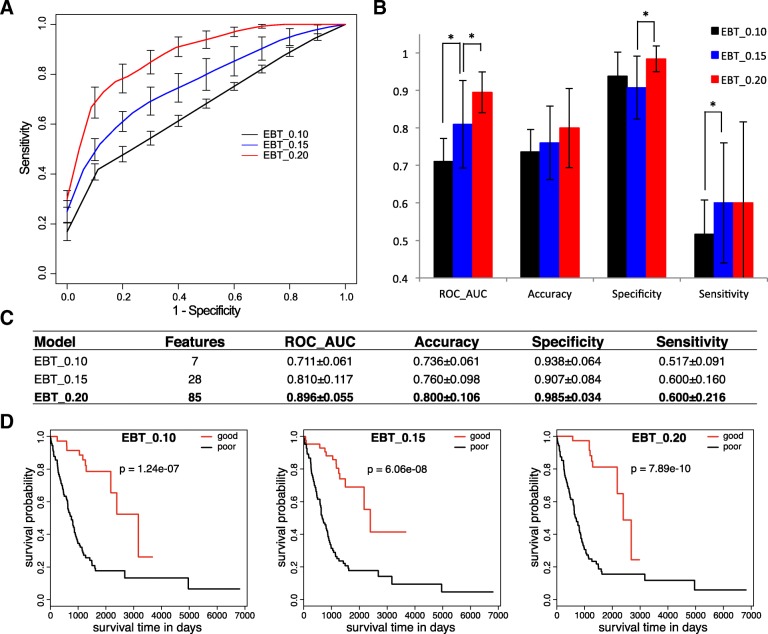
Model performance on prediction of LUAD prognosis based on somatic mutation features. **a**. The ROC curves of SVM models based on different sizes of somatic mutation features. The curves were based on the prediction results of 5-fold cross-validation testing datasets. **b**. Performance comparison of different genetic models. The 5-fold cross-validation results were compared for AUCs of ROC curves (ROC_AUC), Accuracy, Specificity and Sensitivity. Students’ pairwise t tests were performed. Stars represented being significant: * *p* < 0.05. **c**. Performance of different genetic models. The values for each performance measurement was represented as ‘mean ± standard deviation’. **d**. Survival curves of sub-groups of cases classified with different genetic models**.** The curves were based on the prediction results of 5-fold cross-validation testing datasets, and the standard deviations were shown in error bars. Gehan-Breslow-Wilcoxon test *p* values on the overall survival difference between sub-groups were indicated

It is interesting to observe if the genetic variation difference between the prognostic groups could be used for prediction of LUAD treatment outcomes. We adopted a SVM method with different kernels to predict treatment outcomes based on the genetic variance features. As shown in Fig. 2a-c, with the 7 gene features with EBT *p* value < 0.1 between prognosis groups for somatic mutation rate difference, the SVM model (EBT_0.10) reached an average AUC of 0.71 for the 5-fold cross-validated ROC curves. The average accuracy, specificity and sensitivity reached 73.6, 93.8 and 51.7%, respectively (Fig. 2b-c). Survival analysis on the two categories of LUAD cases classified by the model suggested significantly different prognosis between the groups (Fig. 2d, left; Gehan-Breslow-Wilcoxon test, *p* = 1.24e-7).

Two other models (EBT_0.15 and EBT_0.20) were trained with 28 and 85 genes whose mutation rates were significantly different between the good and poor prognostic groups at significance level of EBT *p* < 0.15 and 0.20, respectively. The two models appeared to outperform EBT_0.10 strikingly and model performance was improved when more features (mutated genes) were included (Fig. 2a). The AUC of ROC curve of EBT_0.20 was significantly higher than that of EBT_0.15 (0.896 vs. 0.810, Students’ T test, *p* = 0.044), while the latter model also outperformed EBT_0.10 significantly (0.810 vs. 0.711, *p* = 0.049) (Fig. 2b). EBT_0.20 also showed the highest accuracy (80.0%), specificity (98.5%) and sensitivity (60%) (Fig. 2b-c). The survival curves of cases within either predicted groups of the corresponding model were always differentiated significantly for prognosis, with a strikingly increase of the difference significance for EBT_0.10, EBT_0.15 to EBT_0.20 (Fig. 2d).

The results together suggested an association between the prognosis of LUAD and somatic gene mutations, and the genetic variance could be useful for prognosis prediction.

### Better performance of LUAD prognosis prediction model based on somatic gene mutation features than that based on clinical staging information

**Fig. 3:**
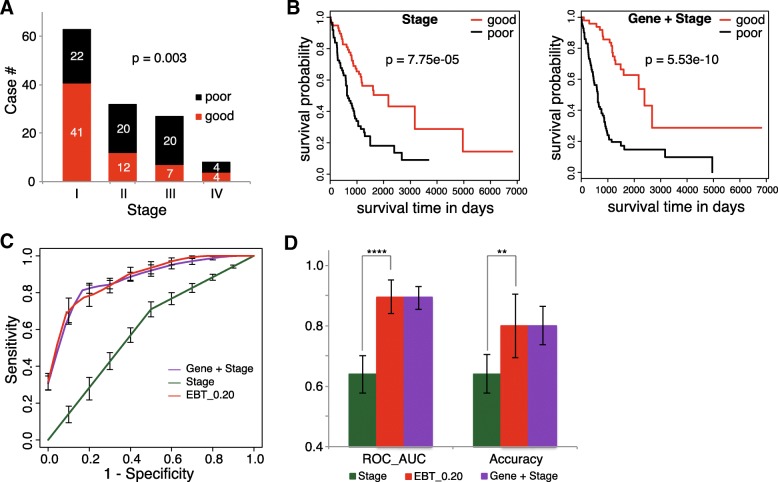
Performance comparison of the prognosis prediction models based on somatic mutation features and clinical stage information. **a**. Correlation between clinical TNM stages and LUAD prognosis. The bars represented different clinical stages while the height of bars represented corresponding case numbers. For each stage, the good and poor prognosis subgroups were shown in different colors. **b**. Survival curves of sub-groups of cases classified with SVM models based on stage (left) or combined stage and somatic mutation features (right). The curves were based on the prediction results of 5-fold cross-validation testing datasets. **c**. The ROC curves of SVM models based on stage, somatic mutation features and the combined features. The curves were based on the prediction results of 5-fold cross-validation testing datasets. **d**. Performance comparison of different genetic models. The 5-fold cross-validation results were compared for ROC_AUC and Accuracy, and the standard deviations were shown in error bars. Students’ pairwise t tests were performed. ** and **** represented *p* < 0.01 and *p* < 0.001, respectively

TNM-based clinical staging system was widely used for LUAD prognosis assessment. The TCGA LUAD cases with staging information were also evaluated for the relationship between stage and prognosis (Additional file 1: Table S3). A significant association was observed, with more poorly prognostic cases at later stages (II and later) (Fig. 3a; Chi-square test, *p* = 0.003). An SVM model was trained only based on stage information, by which the cases were classified into two groups with significantly different prognosis (Fig. 3B, left; Gehan-Breslow-Wilcoxon test, *p* = 7.75e-5). The significance, however, was not comparable to the gene-based models, i.e., EBT_0.10, EBT_0.15 and EBT_0.20 (Fig. 2d). A mixed model was built with combined features of 85 genes (EBT_0.20) and the stage information, and it could also classify the cases into two prognostic groups with higher significance than that of the pure stage model (Fig. 3b, right; *p* = 5.53e-10). A direct comparison of the three models (stage, EBT_0.20 and mixed model) suggested that there was no performance difference between the genetic (EBT_0.20) model and the mixed model, but both outperformed the only stage-based model in terms of ROC-AUC and accuracy (Fig. 3c-d).

### Inter-stage prognosis prediction of the LUAD somatic gene mutation models

It is interesting to observe the gene mutation rate difference between different prognostic LUAD cases at different clinical stages. However, the small size of total samples limited the resolution of stage stratification. Here, the LUAD cases were only stratified into two groups according to their stages, with the ones at Stage I into the early group and the others into the later group (Additional file 1: Table S3). Such a simplified stratification separated all the cases into two groups with nearly identical size (early – 63, later – 67; Fig. 3a). The gene mutation rates were compared between sub-groups with good (≥ 3 years) and poor (< 3 years) prognosis in either early or later group (Additional file 1: Table S4-S5).

**Fig. 4:**
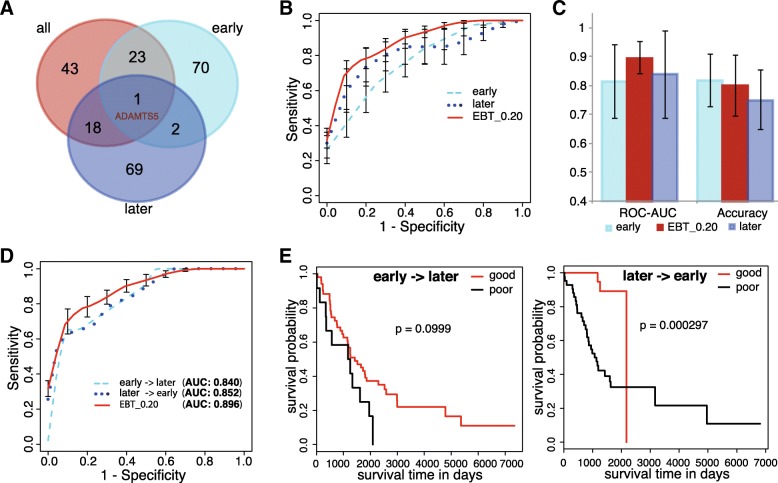
Inter-stage prediction of LUAD prognosis with the genetic models based on somatic mutation features. **a**. Overlap of the top genes with mutation rate difference between good and poor sub-groups for patients diagnosed at all, early and later stages. The name was shown for the gene shared by all the three stratifications. **b**. The ROC curves of SVM models based on different sizes of somatic mutation features. The curves were based on the prediction results of 5-fold cross-validation testing datasets. **c**. Performance comparison of different genetic models. The 5-fold cross-validation results were compared for ROC_AUC and Accuracy, and the standard deviations were shown in error bars. Students’ pairwise t tests were performed. **d**. The inter-stage predictive ROC curves of genetic models. The ‘early -> later’ represented prediction of later patients with the model trained with early cases while the ‘later -> early’ represented prediction of early patients with the model trained with later cases. **e**. Survival curves of sub-groups of cases classified with different inter-stage models

The small size of samples in each group and sub-group led to the much lower statistic power, and much more fewer significant genes were detected at the same significance cutoff as selected for the non-stage-stratified ‘all’ cases. Consequently, a similar number of top genes of smallest *p* values with EBT_0.20 for ‘all’ cases were identified for either group, and compared between each other as well as those for the ‘all’ cases (EBT_0.20). As shown in Fig. 4a, the early group shared 24 genes while the later group shared the similar number of genes (19) with EBT_0.20 for ‘all’ cases. However, only 3 genes were shared between the early and later groups (Fig. 4a). The low consistence of genes with mutation rate difference between prognosis groups could mainly be attributed to the low statistic power and lack of robustness caused by small sample size. Shared by the significant gene sets identified from early, later and ‘all’ group, the only gene, ADAMTS5, could represent an important and stable prognosis factor (Fig. 4a).

Models with the same gene features (EBT_0.20) were also trained and tested with the samples at either stage group. Compared with the EBT_0.20 model, both the early and later models were slightly inferior to EBT_0.20, the model for cases without stage stratification (Fig. 4b). However, the performance difference was not significant (Fig. 4c). The average prognosis prediction accuracy of the early model was even higher than that of EBT_0.20 (Fig. 4c).

To further demonstrate the potential inter-stage application of the 85-gene model, a model was trained with the early cases and used to classify the later cases. Similarly, another model was trained with later cases and classified the early ones. As shown in Fig. 4d, the performance of either model appeared no apparent difference with that of EBT_0.20. The predicted two groups of later cases with early model or those of early cases with later model still showed significant or marginally significant different prognosis (Fig. 4e).

Taking together, the results suggested that the 85-gene model (EBT_0.20) could be applied to LUAD prognosis prediction independent of clinic stage.

## Discussion

In this study, we made a genome-wide somatic mutation profile comparison between different prognosis of LUAD patients. A batch of genes was identified for which the mutation frequencies were strikingly different between prognosis groups. Interestingly, most genes showed higher mutation frequency in the better prognostic group (Fig. 1c and d), indicating the mutations could be benign and beneficial for prognosis. Recently, high tumor mutation burden (TMB) was found associated with better immunotherapy prognosis and was used as an important screening marker for immunotherapy guidance [27, 28]. Gastric cancer genome studies also classified the cases with high gene mutation rates as a major molecular subtype, which often showed better prognosis [29, 30]. More mutations could generate more neo-antigens, which would activate patients’ immune system and consequently increase survival [31, 32]. Functional enrichment analysis suggested that a substantial subset of the mutated genes were related with cell-cell adhesion or cell motility (Fig. 1d). Both adhesion and cell motility are closely related with metastasis [33, 34]. However, further bioinformatic and experimental investigations are needed to confirm whether the genes are associated with tumor prognosis, whether the gene mutations are functional and interacting, and how the mutations could improve prognosis.

Machine-learning models using gene mutation features could well predict LUAD prognosis. Model performance turned better as more genes were included. Even the 7-gene model appeared superior to clinical TNM staging system in prognosis prediction while the 85-gene model performed much better (Figs. 2 and 3). Combination of clinical stage information did not improve the performance of gene models, indicating the independence of somatic gene mutations and clinical stage contributing to LUAD prognosis. However, the genes with most apparent mutation rate difference between good and poor prognosis sub-group showed very few overlaps between early and later cases (Fig. 4a). The extreme sparseness of cases in most of the sub-groups could have led to the low statistic power, precision and therefore the inconsistency. With the 85 featured genes that were identified as the most significant features for non-stage-stratified all samples, models were re-trained only with either early or later cases, and both 5-fold cross validations and inter-stage evaluations suggested the good performance of genetic models independent of clinical stages (Fig. 4b-e). There was one gene consistently identified as one of the genes with most significantly different mutation rates between prognostic (sub)groups, ADAMTS5, whose expression was reported to be correlated with the invasiveness or patient survival of lung and colorectal cancers [35, 36]. As the size of sample increases, more stage-independent genes associated with LUAD prognosis could be identified, and the prognosis prediction would be further improved.

Currently, TNM staging system still plays a central role in LUAD prognosis, though there have been several panels of molecular markers identified for higher prognosis prediction accuracy [37–40]. Recent researches mainly identified markers at the transcription level, including mRNAs, microRNAs or lncRNAs [37, 38]. One of the best-performing panels used 31 lncRNAs and reached 0.881 for the AUC of ROC curves [37]. Our model with genetic markers reached similar or slightly higher AUC (0.896). Compared with RNA (or possibly protein) markers, genetic mutations are qualitative rather than quantitative features and therefore more stable, sensitive, easily and objectively detected. During the revision stage of our manuscript, Cho et al. published a similar study that identified six genetic polymorphism signatures being associated with LUAD prognosis [41]. The authors used classification-oriented feature selection methods to identify most informative mutated genes. Prognosis association analysis was performed to individual genes that were selected as the most relevant features. The best model was reported with ~ 0.88 accuracy, but the ROC curves and AUCs were unknown. None of the feature gene list, procedure for stratification on raw data and optimization strategies for machine learning algorithms was provided, and we could not make a direct comparison. The prognosis prediction effect (accuracy, precision, etc.) was not evaluated on the six genes associated with LUAD prognosis. Among the six genes, MMRN2 was also used as one feature gene in our model (*P* = 0.13, EBT), yet the remained five genes did not show apparent mutation rate difference between prognosis groups in our study (Additional file 1: Table S2). However, Cho et al. and our current study both found the association of genetic mutations and LUAD prognosis independently, and suggested the possible application of these genetic features in clinical guidance of LUAD prognosis.

There are still a couple of drawbacks impeding the application of current prognosis markers. First, larger size of samples with both sequenced genomes and detailed survival follow-up data were needed for refinement of the panels. Secondly, more independent datasets including larger size of patients at different stages are in need to further evaluate the generalization performance of the models. Moreover, for each panel, the tumor tissue will be the major examined material. In practice, however, blood samples could be feasible and convenient to be collected in a noninvasive way. Technique advances in capture and enrichment of circulating tumor cells (CTC) and circulating tumor DNA (ctDNA) makes the blood tests of the prognosis genes promising [42].

## Conclusions

In this research, the somatic gene mutations and prognostic data of TCGA LUAD patients were analyzed. Genes were found with higher mutation rates in good (≥ 3-year survival) than in poor (< 3-year survival) prognosis group. Genes participating in cell-cell adhesion and motility were significantly enriched in the top gene list with mutation rate difference between the good and poor prognosis group of LUAD cases. Machine-learning models with the gene somatic mutation features could well predict LUAD prognosis, and the performance improved as feature size increased. The 85-gene model reached a 5-fold cross-validated ROC-AUC of 0.896, much higher than the widely adopted TNM staging system. The model also exhibited good inter-stage prognosis prediction performance. The genetic features could be used as biomarkers for effective LUAD prognosis prediction.

## Additional file

Additional file 1: **Table S1.** 3-year survival data and classification. **Table S2.** Comparison of gene somatic mutation rates between good and poor prognosis groups. **Table S3.** TNM staging information of LUAD cases with both survival and genome data. **Table S4.** Comparison of gene somatic mutation rates between good and poor prognosis sub-groups of LUAD cases diagnosed at early stage. **Table S5.** Comparison of gene somatic mutation rates between good and poor prognosis sub-groups of LUAD cases diagnosed at later stage. (XLSX 912 kb)

## Abbreviations

AUC: Area Under the Curve
FDR: False Discovery Rate
LUADs: lung adenocarcinomas
NSCLC: Non–small cell lung cancer
ROC: Receiver Operating Characteristic
SVM: Support Vector Machine
TCGA: The Cancer Genome Atlas
TNM: tumor-nodal-metastasis
WGS: whole-genome sequencing

## References

[1] Bray F, Ferlay J, Soerjomataram I, Siegel RL, Torre LA, Jemal A. Global cancer statistics 2018: GLOBOCAN estimates of incidence and mortality worldwide for 36 cancers in 185 countries. CA Cancer J Clin. 2018. 10.3322/caac.21492. 10.3322/caac.21492 30207593

[2] Yang L, Zheng R, Wang N, Yuan Y, Liu S, Li H, Zhang S, Zeng H, Chen W (2018). Incidence and mortality of stomach cancer in China, 2014. Chin J Cancer Res.

[3] Travis WD, Garg K, Franklin WA, Wistuba II, Sabloff B, Noguchi M, Kakinuma R, Zakowski M (2005). Evolving concepts in the pathology and computed tomography imaging of lung adenocarcinoma and bronchioloalveolar carcinoma. J Clin Oncol.

[4] Govindan R, Ding L, Griffith M (2012). Genomic landscape of non-small cell lung Cancer in smokers and never smokers. Cell..

[5] Lynch TJ, Bell DW, Sordella R, Gurubhagavatula S, Okimoto RA, Brannigan BW, Harris PL, Haserlat SM, Supko JG, Haluska FG (2004). Activating mutations in the epidermal growth factor receptor underlying responsiveness of non-small-cell lung cancer to gefitinib. N Engl J Med.

[6] Kohno T, Ichikawa H, Totoki Y, Yasuda K, Hiramoto M, Nammo T, Sakamoto H, Tsuta K, Furuta K, Shimada Y (2012). KIF5B-RET fusions in lung adenocarcinoma. NatMed..

[7] Lipson D, Capelletti M, Yelensky R, Otto G, Parker A, Jarosz M, Curran JA, Balasubramanian S, Bloom T, Brennan KW (2012). Identification of new ALK and RET gene fusions from colorectal and lung cancer biopsies. NatMed..

[8] Soda M, Choi YL, Enomoto M, Takada S, Yamashita Y, Ishikawa S, Fujiwara S, Watanabe H, Kurashina K, Hatanaka H (2007). Identification of the transforming EML4-ALK fusion gene in non- small-cell lung cancer. Nature..

[9] Takeuchi K, Soda M, Togashi Y, Suzuki R, Sakata S, Hatano S, Asaka R, Hamanaka W, Ninomiya H, Uehara H (2012). RET, ROS1 and ALK fusions in lung cancer. Nat Med.

[10] Edge SB, Byrd DR, Compton CC (2009). American joint committee on Cancer Cancer staging manual.

[11] Jemal A, Siegel R, Ward E, Murray T, Xu J, Smigal C, Thun MJ (2006). Cancer statistics, 2006. CA Cancer J Clin.

[12] Sun S, Schiller JH, Spinola M, Minna JD (2007). New molecularly targeted therapies for lung cancer. J Clin Invest.

[13] Lee MC, Kadota K, Buitrago D, Jones DR, Adusumilli PS (2014). Implementing the new IASLC/ATS/ERS classification of lung adenocarcinomas: results from international and Chinese cohorts. J Thorac Dis.

[14] Yoshizawa A, Motoi N, Riely GJ (2011). Impact of proposed IASLC/ATS/ERS classification of lung adenocarcinoma: prognostic subgroups and implications for further revision of staging based on analysis of 514 stage I cases. Mod Pathol.

[15] Paez JG (2004). EGFR mutations in lung cancer: correlation with clinical response to gefitinib therapy. Science..

[16] Pesta M, Kulda V, Fiala O (2012). Prognostic significance of ERCC1, RRM1 and BRCA1 in surgically-treated patients with non-small cell lung Cancer. Anticancer Res.

[17] Drilon A (2013). Response to cabozantinib in patients with RET fusion-positive lung adenocarcinomas. Cancer Discov.

[18] Pleasance ED, Stephens PJ, O'Meara S, McBride DJ, Meynert A, Jones D, Lin ML, Beare D, Lau KW, Greenman C (2010). A small-cell lung cancer genome with complex signatures of tobacco exposure. Nature..

[19] Meyerson M, Gabriel S, Getz G (2010). Advances in understanding cancer genomes through second-generation sequencing. Nat Rev Genet.

[20] Ross JS, Cronin M (2011). Whole cancer genome sequencing by next-generation methods. Am J Clin Pathol.

[21] Mardis ER (2011). A decade's perspective on DNA sequencing technology. Nature..

[22] Han JY, Lee YS, Kim BC, Lee GK, Lee S, Kim EH, Kim HM, Bhak J (2014). Whole-genome analysis of a patient with early-stage small-cell lung cancer. Pharmacogenomics J.

[23] Imielinski M, Berger AH, Hammerman PS (2012). Mapping the hallmarks of lung adenocarcinoma with massively parallel sequencing. Cell..

[24] Wagle N, Berger MF, Davis MJ (2012). High-throughput detection of actionable genomic alterations in clinical tumor samples by targeted, massively parallel sequencing. Cancer Discov..

[25] The Cancer Genome Atlas Research Network (2014). Comprehensive molecular profiling of lung adenocarcinoma. Nature..

[26] Hui X, Hu Y, Sun MA, Shu X, Han R, Ge Q, Wang Y (2017). EBT: a statistic test identifying moderate size of significant features with balanced power and precision for genome-wide rate comparisons. Bioinformatics..

[27] Le DT, Uram JN, Wang H, Bartlett BR, Kemberling H, Eyring AD, Skora AD, Luber BS, Azad NS, Laheru D, Biedrzycki B, Donehower RC, Zaheer A, Fisher GA, Crocenzi TS, Lee JJ, Duffy SM, Goldberg RM, de la Chapelle A, Koshiji M, Bhaijee F, Huebner T, Hruban RH, Wood LD, Cuka N, Pardoll DM, Papadopoulos N, Kinzler KW, Zhou S, Cornish TC, Taube JM, Anders RA, Eshleman JR, Vogelstein B, Diaz LA (2015). PD-1 blockade in tumors with mismatch-repair deficiency. N Engl J Med.

[28] Yarchoan M, Hopkins A, Jaffee EM (2017). Tumor mutational burden and response rate to PD-1 inhibition. N Engl J Med.

[29] Network CGAR (2014). Comprehensive molecular characterization of gastric adenocarcinoma. Nature..

[30] Cristescu R, Lee J, Nebozhyn M, Kim KM, Ting JC, Wong SS, Liu J, Yue YG, Wang J, Yu K, Ye XS, Do IG, Liu S, Gong L, Fu J, Jin JG, Choi MG, Sohn TS, Lee JH, Bae JM, Kim ST, Park SH, Sohn I, Jung SH, Tan P, Chen R, Hardwick J, Kang WK, Ayers M, Hongyue D, Reinhard C, Loboda A, Kim S, Aggarwal A (2015). Molecular analysis of gastric cancer identifies subtypes associated with distinct clinical outcomes. Nat Med.

[31] Brown SD, Warren RL, Gibb EA, Martin SD, Spinelli JJ, Nelson BH, Holt RA (2014). Neo-antigens predicted by tumor genome meta-analysis correlate with increased patient survival. Genome Res.

[32] Rooney MS, Shukla SA, Wu CJ, Getz G, Hacohen N (2015). Molecular and genetic properties of tumors associated with local immune Cytolytic activity. Cell..

[33] Hale JS, Li M, Lathia JD (2012). The malignant social network: cell-cell adhesion and communication in cancer stem cells. Cell Adhes Migr.

[34] Stuelten CH, Parent CA, Montell DJ (2018). Cell motility in cancer invasion and metastasis: insights from simple model organisms. Nat Rev Cancer.

[35] Gu J, Chen J, Feng J, Liu Y, Xue Q, Mao G, Gai L, Lu X, Zhang R, Cheng J, Hu Y, Shao M, Shen H, Huang J (2016). Overexpression of ADAMTS5 can regulate the migration and invasion of non-small cell lung cancer. Tumour Biol.

[36] Li J, Liao Y, Huang J, Sun Y, Chen H, Chen C, Li S, Yang Z (2018). Epigenetic silencing of ADAMTS5 is associated with increased invasiveness and poor survival in patients with colorectal cancer. J Cancer Res Clin Oncol.

[37] Shi X, Tan H, Le X, Xian H, Li X, Huang K, Luo VY, Liu Y, Wu Z, Mo H, Chen AM, Liang Y, Zhang J (2018). An expression signature model to predict lung adenocarcinoma-specific survival. Cancer Manag Res.

[38] Li YY, Yang C, Zhou P, Zhang S, Yao Y, Li D. Genome-scale analysis to identify prognostic markers and predict the survival of lung adenocarcinoma. J Cell Biochem. 2018. 10.1002/jcb.27144. 10.1002/jcb.27144 30105759

[39] Wan YW, Sabbagh E, Raese R, Qian Y, Luo D, Denvir J, Vallyathan V, Castranova V, Guo NL (2010). Hybrid models identified a 12-gene signature for lung Cancer prognosis and Chemoresponse prediction. PLoS One.

[40] Wan YW, Beer DG, Guo NL (2012). Signaling pathway-based identification of extensive prognostic gene signatures for lung adenocarcinoma. Lung Cancer.

[41] Cho HJ, Lee S, Ji YG, Lee DH (2018). Association of specific gene mutations derived from machine learning with survival in lung adenocarcinoma. PLoS One.

[42] Wu X, Zhu L, Ma PC. Next-generation novel noninvasive Cancer molecular diagnostics platforms beyond tissues. Am Soc Clin Oncol Educ Book. 2018;(38):964–77. 10.1200/EDBK_199767 PMC6381937 30231325

